# The impact of HIV infection on skeletal maturity in peripubertal children in Zimbabwe: a cross-sectional study

**DOI:** 10.1186/s12887-024-04965-y

**Published:** 2024-07-27

**Authors:** Farirayi Kowo-Nyakoko, Celia L. Gregson, Leo D. Westbury, Tafadzwa Madanhire, Amaka C. Offiah, Lisa K. Micklesfield, Rashida Abbas Ferrand, Andrea M. Rehman, Kate A. Ward

**Affiliations:** 1 MRC Lifecourse Epidemiology Centre, University of Southampton, Southampton, UK; 2 The Health Research Unit Zimbabwe, Biomedical Research and Training Institute, 10, Seagrave Road, Avondale, Zimbabwe; 3 Department of Medical Physics and Imaging Sciences, University of Zimbabwe-FMHS, Harare, Zimbabwe; 4 Musculoskeletal Research Unit, Bristol Medical School, University of Bristol, Bristol, UK; 5 SAMRC/Wits Developmental Pathways for Health Research Unit, Department of Paediatrics, School of Clinical Medicine, University of the Witwatersrand, Johannesburg, South Africa; 6 Department of Infectious Disease Epidemiology, Faculty of Epidemiology and Population Health, London School of Hygiene and Tropical Medicine, London, UK; 7 Division of Clinical Medicine, University of Sheffield, Sheffield, UK; 8 Clinical Research Department, Faculty of Infectious and Tropical Diseases, London School of Hygiene and Tropical Medicine, London, UK; 9 MRC International Statistics and Epidemiology Group, Department of Infectious Disease Epidemiology, Faculty of Epidemiology and Population Health, London School of Hygiene and Tropical Medicine, London, UK; 10 MRC Unit @ London School of Hygiene and Tropical Medicine, Banjul, The Gambia

**Keywords:** Adolescence, Africa, Bone age, Children, HIV, Puberty

## Abstract

**Introduction:**

HIV infection and its treatment compromises skeletal development (growth and maturation). Skeletal maturity is assessed as bone age (BA) on hand and wrist radiographs. BA younger than chronological age (CA) indicates delayed development. We conducted a cross-sectional study to determine differences between BA and CA (i.e., skeletal maturity deviation [SMD]), and risk factors associated with SMD in peripubertal children with and without HIV established on antiretroviral therapy (ART) including use of tenofovir disoproxil fumarate (TDF).

**Methods:**

Children with HIV taking ART for at least two years and a comparison group of HIV-negative children, aged 8–16 years and frequency-matched by age and sex, were recruited from HIV clinics and local schools in the same catchment area, in Harare, Zimbabwe. BA was assessed from non-dominant hand-wrist radiographs using the Tanner Whitehouse 3 method. Negative SMD values correspond to delayed development, i.e., BA younger than CA. Multivariable linear regression models determined factors associated with SMD overall, and in children with HIV.

**Results:**

In total, 534 participants (54% males) were included; by design CA was similar in males and females, whether living with or without HIV. Mean (SD) SMD was more negative in CWH than in HIV-negative children in both males [-1.4(1.4) vs. -0.4(1.1) years] and females [-1.1(1.3) vs. -0.0(1.2) years]. HIV infection and weight-for-age Z-score<-2 were associated with more negative SMD in both males and females after adjusting for socio-economic status, orphanhood, pubertal stage, and calcium intake. Age at ART initiation was associated with SMD in both males and females with those starting ART later more delayed: starting ART aged 4–8 years 1.14 (-1.84, -0.43), or over 8 years 1.47 (-2.30, -0.65) (*p*-value for trend < 0.001). Similar non-significant trends were seen in males. TDF exposure TDF exposure whether < 4years or ≥ 4 years was not associated with delayed development.

**Conclusion:**

Perinatally-acquired HIV infection and being underweight were independently associated with delayed skeletal maturation in both males and females. Starting ART later was independently associated with skeletal maturation delay in CWH. Given the known effects of delayed development on later health, it is important to find interventions to ensure healthy weight gain through early years and in CWH to initiate ART as early as possible.

**Supplementary Information:**

The online version contains supplementary material available at 10.1186/s12887-024-04965-y.

## Introduction

In 2022, 39 million people were living with HIV worldwide; two-thirds in sub-Saharan Africa [1]. The roll-out of antiretroviral therapy (ART) has resulted in a remarkable increase in life-expectancy, but coverage of treatment substantially lags in children: in 2022, 76% of adults versus 57% of children (aged up to 14 years) were accessing ART.

While increasing numbers of children with HIV (CWH) are reaching adulthood because of ART, longstanding HIV infection and/or treatment is associated with an increased risk of multisystem morbidities, including an adverse effect on development, i.e. growth and maturation [2, 3]. One of the first recognised manifestations of perinatally-acquired HIV infection was poor linear growth [2]. Additionally, HIV infection has been associated with low bone mineral density and impaired bone architecture [3–6]. Delays in development can have consequences on an individual’s health in later life, as well as intergenerational effects. On average, during adolescence, individuals gain 20% of their final height and 50% of their body mass with considerable remodelling of the skeleton during adolescence making it a critical period for health [7].

Skeletal maturity is a measure of development incorporating the size, shape, and degree of mineralisation of the epiphyses and physeal plates of bone to define their proximity to full maturity. Maturation is a sequence of changes through growth and puberty which culminates in the development of secondary sexual characteristics and cessation of linear growth [8]. Bone age (BA) is an objective measure of skeletal maturation and is assessed by hand-wrist radiographs. The bones of the hand and wrist mature sequentially, and the stages of this process can be compared against a reference standard. A lag of BA behind chronological age (CA) (calculated from the date of birth) indicates impaired skeletal development in children. Skeletal maturation is influenced by several factors such as genetic ancestry and environmental factors like socio-economic deprivation, nutritional status (e.g., vitamin D and calcium intake), physical activity and co-morbid disease [4, 6, 9]. Studies have suggested that CWH may have delayed skeletal maturation despite ART [10, 11]; however, none have been conducted in sub-Saharan Africa where the majority of CWH live. Given the impact of maturation on achieving genetic potential and consequently future health, it is important to understand how skeletal development progresses through puberty in males and females from sub-Saharan Africa. This study in peripubertal males and females from Zimbabwe, therefore, aimed to describe the differences between BA and CA (i.e., skeletal maturity deviation [SMD]), determine to what extent HIV and other demographic and lifestyle factors predict SMD, and examine which HIV characteristics are associated with SMD in children living with HIV in Zimbabwe.

## Methods

### Study design and participants

A cross-sectional study of children aged 8–16 years, with and without HIV was conducted in Harare, Zimbabwe, nested within the IMpact of Vertical HIV infection on child and Adolescent Skeletal development (IMVASK) study; the protocol has previously been published [3]. Zimbabwe has experienced an early inset, sustained generalised HIV epidemic with an adult HIV prevalence of 11% in 2022. Of the 1.3 million people living with HIV, 75 000 are children [12]. CWH were recruited from HIV clinics at the two main public sector (tertiary referral) hospitals in Harare (Parirenyatwa and Sally Mugabe Hospital) between May 4, 2018, and Jan 21, 2020. Both hospitals have paediatric HIV clinics that provide HIV care and treatment to more than 2,000 children [3]. Although HIV care is increasingly decentralised to a primary care level across the country, most children in Harare continue to receive care within the HIV clinics in these healthcare facilities. Individuals were eligible if they had been taking ART for at least two years, were not acutely unwell, were residing in Harare and aware of their HIV status. Systematic quota-based sampling was used to recruit children stratified by sex into three age groups (8–10, 11–13 and 14–16 years). HIV-negative children were recruited from six public-sector schools within the same catchment area as the hospitals, again by stratified random sampling using school registers [3].

Boys and girls with CA above 16.5 and 15 years respectively were excluded for this analysis as these are above the cut off ages for Tanner Whitehouse 3 method of BA assessment.

### Procedures

Sociodemographic and clinical data were collected by trained research staff using an interview-administered questionnaire. Data were collected on android tablets using the Online Data Kit (https://getodk.org/). A nurse and/or doctor carried out Tanner pubertal staging using testicular volume (assessed using an orchidometer), penile size and pubic hair growth (quality distribution and length) in boys and breast development (size and contour), age at menarche and pubic hair growth in girls for assessment [13]. Grading of penile, testicular and breast growth was from I to V as per Tanner descriptions [14–16]. In the event of discordance in the assignment of the pubertal stage between these categories, testicular and breast development for boys and girls respectively, were used to assign Tanner stage. Socio-economic status (SES) was constructed as three groups (low, middle, and high) using the first component from a principal component analysis that included the head of household age, highest maternal and paternal education levels, monthly household income, number in the household, household ownership, access to amenities and household asset ownership [17]. Dietary calcium and vitamin D intake were quantified using a validated dietary diversity and food frequency tool from India and Malawi, adapted to the Zimbabwean context [18]. Daily calcium dietary intake was classified into three groups: very low (< 150 mg/day), low (150–299 mg/day), and moderate (300–450 mg/day). Daily dietary vitamin D intake was classified as very low (< 4.0 μg/day), low (4.0–5.9 μg/day), and moderate (6.0–8.0 μg/day) [19]. These thresholds were determined based on the distribution of intakes across the population [4]. Physical activity was self-reported using the International Physical Activity Questionnaire which has been validated in multiple countries; this assessment was based on multiples of the resting metabolic rate in MET minutes/week and categorised as low (< 600 MET minutes/week), moderate (600–3000 MET minutes/week), and high (> 3000 MET minutes/week) physical activity [20].

For participants with HIV, ART regimen including use of tenofovir disoproxil fumarate (TDF) was recorded; CD4 count was measured using an Alere PIMA CD4 analyser (Waltham, MA, USA). A GeneXpert HIV-1 viral load platform (Cepheid, Sunnyvale, CA, USA) was used to measure HIV viral load.

### Anthropometric measurements

Standing height and weight were measured in duplicate by two independent trained staff members (nurse and research assistant). If the two measurements differed by more than 0.5 kg–0.5 cm, a third reading was taken by an additional reader. The mean of the two or three measurements was recorded as the final figure. Weight was measured to the nearest 0.1 kg using a Seca 875 weight scale, and height to the nearest 0.1 cm using a stadiometer (Seca, Hamburg Germany).

### Bone age assessment

Digital hand-wrist radiographs of the non-dominant side were taken by a trained radiographer. Details of the method of obtaining the radiographs have previously been published [21]. BA was assessed using the Tanner Whitehouse 3 Radius, Ulna and Short bones method which has been found to be precise and, in contrast to the Greulich-Pyle method, not biased by age in this population of peripubertal adolescents [21, 22].

### Statistical analysis

Data were analysed using Stata 17 (StatCorp, TX, USA). Weight for age z-scores (WAZ), Height for age z-scores (HAZ) and body mass index (BMI) for age z-scores were calculated using the 1990 UK reference data for children [23], because World Health Organization reference data for WAZ are not available beyond the age of 10 years [24]. Z-scores of -2.0 or lower for HAZ, WAZ and BMI z-score were used to define stunting, underweight and wasting respectively [25]. Analyses were stratified by sex because BA assessment and bone development are sex specific. The primary outcome was SMD in years. A negative SMD value reflects a delay in skeletal maturity, and a positive value reflects advanced skeletal maturity relative to CA.

Quantitative data were examined for normality using the Shapiro–Wilk test and histograms. Normally distributed continuous variables were presented as means ± standard deviations and categorical variables as numbers and proportions of participants in each category. Non-normally distributed continuous variables were presented as medians and interquartile ranges (IQR). Participant characteristics were compared by HIV status, using the student t-test for continuous variables and chi-squared test for categorical variables. To account for missing data on pubertal status, CD4 count and viral load, we first identified auxiliary variables (HIV status, socio-economic status, sex, chronological age, physical activity, vitamin D and calcium intake). These were added to the imputation model to increase power and to support the plausibility of the assumption of missing at random. These auxiliary variables were identified: (1) as an a priori factor; (2) after considering their strength of association with the variables containing missing values (if continuous, correlation coefficient *r* > 0.4 and if categorical, chi squared test *p* < 0.05). Using imputation by chained equations in Stata a binary distribution was used to impute dichotomised CD4 count and viral load whilst an ordinal distribution was used to impute Tanner staging.

Linear regression was performed to examine associations between exposure variables (HIV status, WAZ, socio-economic deprivation, orphanhood, physical activity, vitamin D intake, calcium intake, pubertal stage) and SMD. WAZ was used instead of BMI because of the fixed relationship between skeletal maturation and height, which contributes to the BMI calculation. For ordinal exposures, *p*-values from tests for trend were additionally reported. Firstly, unadjusted linear regression models were used to determine the univariable associations between the exposure variables outlined above and SMD. Secondly, a multivariable linear regression model considered HIV and a priori factors (WAZ, SES, pubertal stage, orphanhood, and calcium intake) as exposures. *A priori factors* were chosen based on previous literature showing that lower WAZ, low SES, orphanhood and low dietary calcium intake are related to other skeletal outcomes [3, 6, 26, 27]. In addition, orphanhood was shown to be associated with low bone density in this cohort [4]. Other variables were included in the multivariable model if there was any evidence of a potential association with SMD in the boys or the girls in the unadjusted analyses (*p*-value ≤ 0.1).

In an analysis restricted to CWH, linear regression was used to assess associations between HIV specific variables (age at ART initiation, CD4 count, viral load and TDF exposure) and SMD. The multivariable linear regression model in CWH included a priori factors outlined above and HIV specific variables with a *p*-value for trend ≤ 0.1 in the univariable linear regression analysis.

Collinearity was assessed using the variance inflation factor with values above 5 indicating collinearity [28]. Standard errors for regression coefficients were also assessed to determine the robustness of models. Regression assumptions were assessed to ensure (1) normality of residuals using Q-Q plots, (2) homogenous variance in scatter plot of fitted values versus residuals, and (3) independence of residuals by examining scatter plots of residuals versus exposure variables.

## Results

### Participant characteristics

**Fig. 1:**
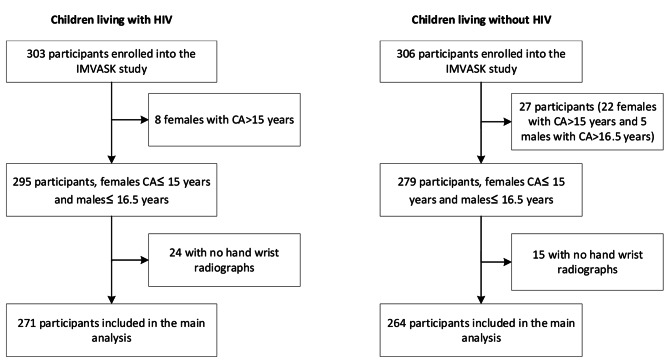
Flow diagram showing the participants included in the analysis. IMVASK: The **Im**pact of **V**ertical HIV infection on child and **A**dolescent **Sk**eletal development, **CA**-chronological age

We initially recruited 609 (303 HIV negative) participants into the study, of whom 74 were excluded ( 35 were above 15 and 16.5 years for females and males respectively and 39 did not have hand-wrist radiographs) with 535 participants (88% of original sample, 54% males) included in the analysis (Fig. 1).

For both males and females, a larger proportion of CWH had lower SES, were orphaned (one or both parent dead), stunted, or underweight in comparison to HIV-negative participants (Table 1). On average, male CWH were 7.1 cm shorter and 3.3 kg lighter than male HIV-negative participants; similarly female CWH were 6.0 cm shorter and 6.4 kg lighter than their HIV-negative peers. Female CWH were more likely to be in earlier Tanner stages (1 and 2) in comparison to those who were HIV-negative, whilst no differences were seen in Tanner stage by HIV status in males. Notably, over 65% of participants had low or very low intakes of calcium, falling far below recommended rates of other populations (e.g., UK recommendations are for calcium intakes between 800 and 1000 mg/day at this age) [29], and there was no difference in intake by HIV status. CWH reported lower levels of physical activity compared to HIV-negative participants.

CWH were diagnosed at a median age of 3.0 years (IQR 1.1–5.9) and were established on ART at 3.8 years (IQR 1.9-7.0). Overall, mean ART duration was 7.6 (SD 2.6) years, and 87 participants were on a TDF regimen with a median duration of 3.0 years (IQR 1.4–5.5). A high proportion of CWH had a suppressed viral load and a CD4 count > 500 cells per microlitre (79% and 80% respectively) [30] (Supplementary Table 1).

**Table 1:** Characteristics of study participants by HIV status in males and females

	Males			Females		
	HIV- (*n* = 141)	HIV+ (*n* = 146)	*p*-value	HIV- (*n* = 123)	HIV+ (*n* = 125)	*p*-value
**Socio-demographics**						
Chronological age (years), mean (SD)	12.3 (2.4)	12.6 (2.5)	0.220	12.1 (2.2)	12.3 (2.4)	0.520
Socio-economic status, n (%)			0.180			0.008
Group 1: low	54 (38.3)	46 (31.5)		51 (41.5)	29 (23.2)	
Group 2: middle	53 (37.6)	49 (33.6)		35 (28.5)	43 (34.4)	
Group 3: high	34 (24.1)	51 (34.9)		37 (30.1)	53 (42.4)	
Orphanhood: One or both parents dead, n (%)	11 (7.9)	59 (42.4)	< 0.001	7 (5.8)	52 (43.0)	< 0.001
**Anthropometry**						
Height (cm), mean (SD)	146.9 (15.1)	139.8 (12.3)	< 0.001	145.4 (11.9)	139.4 (12.9)	< 0.001
Height for age z-score, mean (SD)	-0.6 (0.9)	-1.7 (1.1)	< 0.001	-0.5 (1.1)	-1.5 (1.1)	< 0.001
Stunting (height for age z-score < -2), n (%)	8 (5.7)	52 (35.6)	< 0.001	9 (7.6)	31 (27.0)	< 0.001
Weight (kg), mean (SD)	38.7 (14.5)	35.4 (17.4)	< 0.001	41.6 (16.7)	35.2 (13.5)	< 0.001
Weight for age z-score, mean (SD)	-0.7 (1.1)	-1.6 (1.2)	< 0.001	-0.3 (1.2)	-1.3 (1.2)	< 0.001
Underweight (weight for age z-score < -2), n (%)	15 (10.6)	47 (32.2)	< 0.001	7 (5.7)	27 (21.6)	< 0.001
BMI (kg/m^2^), mean (SD)	17.1(2.2)	16.6(1.5)	0.013	19.1(3.6)	17.5(2.7)	< 0.001
BMI for age z-score, mean (SD)	-0.5 (1.0)	-0.8 (0.9)	0.026	-0.1 (1.2)	-0.6 (0.9)	< 0.001
Wasting (BMI for age z-score < -2), n (%)	11 (7.8)	13 (8.9)	1.000	4 (3.3)	8 (6.4)	0.370
**Pubertal status**			0.240			< 0.001
Tanner I	45 (31.9)	55 (40.1)		23 (19.0)	52 (43.7)	
Tanner II	34 (24.1)	37 (27.0)		30 (24.8)	18 (15.1)	
Tanner III	22 (15.6)	21 (15.3)		28 (23.1)	27 (22.7)	
Tanner IV	36 (25.5)	19 (13.9)		31 (25.6)	18 (15.1)	
Tanner V	4 (2.8)	5 (3.6)		9 (7.4)	4 (3.4)	
**Lifestyle factors**						
Physical activity level, n (%)			0.043			0.055
*Low*,* < 600 MET mins/week*	48 (34.0)	46 (31.5)		44 (35.8)	29 (23.2)	
*Moderate*,* 600–3000 MET mins/week*	47 (33.3)	33 (22.6)		32 (26.0)	30 (24.0)	
*High*,* > 3000 MET mins/week*	46 (32.6)	67 (45.9)		47 (38.2)	66 (52.8)	
Daily vitamin D intake, n (%)			0.910			0.350
*Very low*,* < 4.0 μg/day*	32 (22.7)	27 (18.5)		29 (23.6)	22 (17.6)	
*Low*,* 4.0–5.9 μg/day*	92 (65.2)	96 (65.8)		79 (64.2)	89 (71.2)	
*Moderate*,* 6.0–8.0 μg/day*	17 (12.1)	23 (15.8)		15 (12.2)	14 (11.2)	
Daily calcium intake, n (%)			0.950			0.990
*Very low*,* < 150 mg/day*	48 (34.0)	52 (35.6)		42 (34.1)	44 (35.2)	
*Low*,* 150–299 mg/day*	31 (22.0)	30 (20.5)		27 (22.0)	27 (21.6)	
*Moderate*,* 300–450 mg/day*	62 (44.0)	64 (43.8)		54 (43.9)	54 (43.2)	
**Bone age measures**						
Bone age (years), mean (SD)	11.8 (2.5)	11.2 (2.3)	0.042	12.0 (2.2)	11.2 (2.4)	0.005
Skeletal maturity deviation	-0.4 (1.1)	-1.4 (1.4)	< 0.001	-0.0 (1.2)	-1.1 (1.4)	< 0.001
Skeletal maturity delay, n (%)	11 (7.8)	45 (30.8)	< 0.001	6 (4.9)	26 (20.8)	< 0.001

Student t-tests conducted on continuous variables and chi-squared tests on categorical variable. MET – multiples of the resting metabolic rate SD- Standard deviation. Skeletal maturity deviation-difference between bone age and chronological age. Skeletal maturity delay-skeletal maturity deviation </=-2 year

### SMD in children with and without HIV

**Fig. 2:**
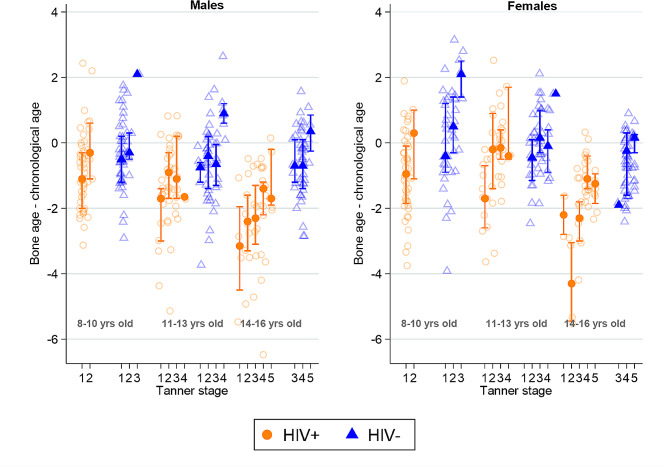
Comparison of mean skeletal maturity deviation (difference between bone age and chronological age) in children living with and without HIV by sex, age group and pubertal status. Error bars indicate 95% confidence intervals

On average, there was little evidence of SMD in the HIV-negative group, particularly in females; however, SMD was evident in CWH whether male or female, such that skeletal maturity was delayed by more than a year compared to HIV-negative participants (Table 1). When stratified by Tanner stage, BA values were much lower than CA values in older children in earlier Tanner stages (Fig. 2).

Among males, living with HIV, being underweight, being orphaned, and consuming less dietary calcium, were associated with a more marked SMD (i.e., more delayed skeletal maturation) in univariable analyses. After adjustment in a multivariable model (which also included SES and pubertal stage), only living with HIV and being underweight remained associated with negative SMD in males (Table 2). In females, before adjustment, living with HIV, being underweight, orphaned, and to a lesser degree earlier pubertal stage was associated with more negative SMD. After adjustment in a multivariable model (which also included dietary calcium and SES), again only living with HIV and being underweight remained associated with greater delay in SMD in females (Table 3).

**Table 2:** Associations between participant characteristics and skeletal maturity deviation (SMD) in males (unadjusted and adjusted linear regression analysis)

Variable	Males *n* = 287			
	Unadjusted model	*p*-value	Adjusted model	*p*-value
	β coefficient (95%CI)		β coefficient (95%CI)	
**HIV status**				
HIV negative	ref	< 0.001	ref	< 0.001
HIV positive	-0.96 (-1.25, -0.66)		-0.59 (-0.90, -0.28)	
**Weight for age z-score**				
Weight for age z-score>-2	ref	< 0.001	ref	< 0.001
Weight for age z-score<-2	-1.41 (-1.83, -1.15)		-1.18 (-1.52, -0.84)	
**Socio-economic status**				
*Group 1: High*	ref	0.742	ref	0.440
*Group 2: Middle*	0.29 (-0.10, 0.68)		0.33 (-0.01, 0.67)	
*Group 3: Low*	-0.08 (-0.31, 0.47)		-0.11 (-0.44, 0.22)	
**Pubertal stage**				
Tanner 1	ref	0.990	ref	0.693
Tanner 2	0.07 (-0.34, 0.48)		0.12 (-0.23, 0.47)	
Tanner 3	-0.05 (-0.53, 0.43)		0.02 (-0.39, 0.43)	
Tanner 4&5	0.02 (-0.40, 0.45)		-0.06 (-0.43, 0.30)	
**Orphan status**				
Not an orphan	Ref	<0.001	ref	0.105
One or both parents dead	-0.76 (-1.11, -0.41)		-0.32 (-0.68, 0.02)	
**Physical activity**				
*High*,* > 3000 MET mins/week*	Ref	0.977		
*Moderate*,*600-3000MET mins/week*	0.37 (-0.02, 0.76)			
*Low*,* < 600 MET mins/week*	-0.03 (-0.39, 0.34)			
**Vitamin D intake**				
*Moderate*,* 6.0–8.0 μg /day*	Ref	0.297		
*Low*,* 4.0–5.9 μg /day*	-0.14 (-0.61, 0.32)			
*Very low*,* < 4.0 μg /day*	0.22 (-0.32, 0.76)			
**Calcium intake**				
*Moderate*,* 300–450 mg/day*	Ref	0.046	ref	0.114
*Low*,* 150–299 mg/day*	0.18 (-0.25, 0.61)		0.16 (-0.20, 0.53)	
*Very low*,* < 150 mg/day*	-0.34 (-0.70, 0.01)		-0.24(-0.55 0.07)	

The adjusted model included the following exposures: HIV status, weight for age z-scores, socio-economic status, pubertal stage, orphan status, and calcium intake. *P*-value for trend shown for variables with more than one category. CI-confidence interval, SMD-Skeletal maturity deviation calculated as the difference between bone age and chronological age; negative values reflect lower bone age than chronological age (delay in skeletal maturation)

**Table 3:** Associations between participant characteristics and skeletal maturity deviation (SMD) in females (unadjusted and adjusted linear regression analysis)

Variable	Females *n* = 246			
	Unadjusted model	*p*-value	Adjusted model	*p*-value
	β coefficient (95%CI)		β coefficient (95%CI)	
**HIV status**				
HIV negative	Ref	< 0.001	ref	< 0.001
HIV positive	-1.03 (-1.35, -0.71)		-0.91 (-1.28, -0.55)	
**Weight for age z-score**				
Weight for age z-score>-2	Ref	< 0.001	ref	< 0.001
Weight for age z-score<-2	-1.64 (-2.10, -1.19)		-1.40 (-1.86, -0.93)	
**Socio-economic status**				
Group 1: High	Ref	0.512	ref	0.054
Group 2: Middle	0.23 (-0.20, 0.65)		-0.004 (-0.38, 0.37)	
Group 3: Low	0.13 (-0.29, 0.55)		-0.38, (-0.76, -0.003)	
**Pubertal stage**				
Tanner 1	Ref	0.053	ref	0.898
Tanner 2	0.42 (-0.09, 0.92)		0.01 (-0.45, 0.47)	
Tanner 3	0.68 (0.21, 1.16)		0.38 (-0.05, 0.80)	
Tanner 4&5	0.37 (-0.09, 0.83)		-0.17 (-0.60, 0.27)	
**Orphan status**				
Not an orphan	ref	0.003	ref	0.914
One or both parents dead	-0.60 (-0.99, -0.20)		-0.04 (-0.45, 0.36)	
**Physical activity**				
High, > 3000 MET mins/week	ref	0.214		
Moderate,600-3000MET mins/week	0.17 (-0.26, 0.60)			
Low, < 600 MET mins/week	0.25 (-0.16, 0.66)			
**Vitamin D intake**				
Moderate, 6.0–8.0 μg /day	ref	0.509		
Low, 4.0–5.9 μg /day	0.12 (-0.43, 0.67)			
Very low, < 4.0 μg /day	0.21 (-0.42, 0.85)			
**Calcium intake**				
Moderate, 300–450 mg/day	ref	0.292	ref	0.881
Low, 150–299 mg/day	0.11 (-0.36, 0.59)		0.19 (-0.23, 0.61)	
Very low, < 150 mg/day	-0.20 (-0.60, 0.19)		-0.01 (-0.37, 0.34)	

The adjusted model included the following exposures: HIV status, weight for age z-scores, socio-economic status, pubertal stage, orphan status, and calcium intake. *P*-value for trend shown for variables with more than one category. CI-confidence interval, SMD-Skeletal maturity deviation calculated as the difference between bone age and chronological age; negative values reflect lower bone age than chronological age (delay in skeletal maturation)

### Factors associated with SMD in children with HIV

There was no difference in physical activity levels, and dietary vitamin D and calcium intake by sex but the mean WAZ and BMI for age z-scores differed with males [*n* = 40 (34%)] more likely to be underweight than females [*n* = 22 (22%)] (Supplementary Table 2). Females had less negative SMD (i.e., less delay in skeletal maturation) than the males.

In univariable analyses, older age at ART initiation (in both sexes) and greater viral load (in males only) were associated with more negative SMD (Tables 4 and 5). After adjustment for WAZ, SES, pubertal stage, orphan status, viral load, and years of TDF exposure, there was negative association between age at ART initiation and SMD and calcium intake and SMD in males and the negative association between viral load and SMD was partially attenuated. No association between viral load and SMD was found in females, but later age at ART initiation was still associated with more negative SMD (Table 5). The association between WAZ and SMD was robust to adjustment, and being underweight was still associated with more negative SMD in CWH in both sexes. Underweight children (z-score < -2) were on average delayed by a year in their skeletal maturity, compared to those who were not underweight (Tables 4 and 5).

**Table 4:** Associations between participant characteristics and skeletal maturity deviation (SMD) in males living with HIV (unadjusted and adjusted linear regression analysis)

Variable	Unadjusted model	*p*-value	Adjusted model	*p*-value
	β coefficient (95%CI)		β coefficient (95%CI)	
**Males ** ***n*** ** = 146**				
**Weight for age z-score**				
Weight for age z-score>-2	ref	< 0.001	ref	0.001
Weight for age z-score<-2	-1.22 (-1.67, -0.77)		-0.92 (-1.41, -0.44)	
**Socio-economic status**				
*Group 1: High*	ref	0.187	ref	0.101
*Group 2: Middle*	-0.06 (-0.62, 0.49)		0.21 (-0.35, 0.78)	
*Group 3: Low*	-0.38 (-0.94, 0.18)		-0.45 (-0.98, 0.08)	
**Pubertal stage**				
Tanner 1	ref	0.115	ref	0.941
Tanner 2	0.04 (-0.53, 0.62)		0.26 (-0.30, 0.82)	
Tanner 3	-0.17 (-0.86, 0.52)		-0.08 (-0.76, 0.61)	
Tanner 4&5	-0.54 (-1.19, 0.12)		0.09 (-0.59, 0.76)	
**Orphan status**				
Not an orphan	ref	0.046	ref	0.298
One or both parents dead	-0.52 (-0.98, -0.06)		-0.27 (-0.73, 0.19)	
**Physical activity**				
*High*,* > 3000 MET mins/week*	ref	0.765		
*Moderate*,* 600–3000 MET mins/week*	0.11 (-0.48, 0.70)			
*Low*,* < 600 MET mins/week*	-0.09 (-0.63, 0.44)			
**Vitamin D intake**				
*Moderate*,* 6.0–8.0 μg /day*	ref	0.187		
*Low*,* 4.0–5.9 μg /day*	-0.08 (-0.72, 0.56)			
*Very low*,* < 4.0 μg /day*	0.49 (-0.29, 1.27)			
**Calcium intake**				
*Moderate*,* 300–450 mg/day*	ref	0.010	ref	0.019
*Low*,* 150–299 mg/day*	0.12 (-0.50, 0.74)		-0.06 (-0.66, 0.54)	
*Very low*,* < 150 mg/day*	-0.65 (-1.15, -0.15)		-0.62 (-1.13, -0.10)	
**Age at ART initiation**				
< 2 years	ref	< 0.001	ref	0.026
2-3.9 years	-0.63 (-1.23, -0.02)		-0.50 (-1.11, 0.11)	
4–8 years	-0.83 (-1.40, -0.25)		-0.73 (-1.33, -0.12)	
> 8 years	-1.28 (-2.01, -0.54)		-0.75 (-1.54, 0.04)	
**CD4 count**				
>/=500 cells per μL	ref	0.191		
< 500 cells per μL	-0.36 (-0.91, 0.18)			
**Viral load**				
< 1000 RNA copies per ml	ref	0.004	ref	0.052
> 1000 RNA copies per ml	-0.85 (-1.43, -0.28)		-0.58 (-1.17, 0.01)	
**TDF years of exposure**				
No exposure	ref	0.052	ref	0.977
< 4 years	-0.43 (-1.01, 0.15)		-0.04 (-0.63, 0.56)	
≥ 4 years	-0.56 (-1.24, 0.11)		0.01 (-0.72, 0.73)	

The adjusted model included the following exposures: pubertal status, weight for age z-scores, socio-economic status, orphan status, calcium intake, age at ART initiation and viral load. ART-Anti-retroviral therapy, TDF- tenofovir disoproxil fumarate. CI-confidence interval SMD: Skeletal maturity deviation calculated as the difference between bone age and chronological age; negative values reflect lower bone age than chronological age (delay in skeletal maturation) *mean SMD for each category

**Table 5:** Associations between participant characteristics and skeletal maturity deviation (SMD) in females living with HIV (unadjusted and adjusted linear regression analysis)

Variable	Unadjusted model	*p*-value	Adjusted model	*p*-value
	β coefficient (95%CI)		β coefficient (95%CI)	
**Females ** ***n*** ** = 125**				
**Weight for age z-score**				
Weight for age z-score>-2	ref	< 0.001	ref	0.001
Weight for age z-score<-2	-1.27 (-1.82, -0.72)		-0.99 (-1.58, -0.40)	
**Socio-economic status**				
*Group 1: High*	ref	0.878	ref	0.460
*Group 2: Middle*	0.37 (-0.19, 0.93)		0.22 (-0.33, 0.76)	
*Group 3: Low*	-0.04 (-0.66, 0.58)		-0.17 (-0.77, 0.44)	
**Pubertal stage**				
Tanner 1	ref	0.221	ref	0.023
Tanner 2	0.10 (-0.65, 0.85)		0.55 (-0.22, 1.32)	
Tanner 3	0.35 (-0.28, 0.99)		0.69 (0.06, 1.32)	
Tanner 4	0.33 (-0.35, 1.01)		0.69 (-0.04, 1.42)	
**Orphan status**				
Not an Orphan	ref	0.920	ref	0.920
One or both parents dead	-0.02 (-0.51, 0.46)		0.09 (-0.41, 0.58)	
**Physical activity**				
*High*,* > 3000 MET mins/week*	ref	0.490		
*Moderate*,* 600–3000 MET mins/week*	-0.01 (-0.61, 0.60)			
*Low*,* < 600 MET mins/week*	0.23 (-0.37, 0.84)			
**Vitamin D intake**				
*Moderate*,* 6.0–8.0 μg /day*	ref	0.427		
*Low*,* 4.0–5.9 μg /day*	0.49 (-0.29, 1.27)			
*Very low*,* < 4.0 μg /day*	0.45 (-0.48, 1.38)			
**Calcium intake**				
*Moderate*,* 300–450 mg/day*	ref	0.133	ref	0.998
*Low*,* 150–299 mg/day*	-0.34 (-1.00, 0.33)		-0.19 (-0.81, 0.43)	
*Very low*,* < 150 mg/day*	-0.42 (-0.97, 0.13)		-0.04 (-0.56, 0.49)	
**Age at ART initiation**				
< 2 years	ref	0.001	ref	< 0.001
2-3.9 years	-0.35 (-0.99, 0.30)		-0.48 (-1.10, 0.15)	
4–8 years	-0.83 (-1.46, -0.19)		-1.21 (-1.88, -0.54)	
> 8 years	-1.02 (-1.69, -0.34)		-1.59 (-2.33, -0.84)	
**CD4 count**				
>/=500 cells per μL	ref	0.660		
< 500 cells per μL	-0.15 (-0.82, 0.52)			
**Viral load**				
< 1000 RNA copies per ml	ref	0.196	ref	0.176
> 1000 RNA copies per ml	0.41 (-0.19, 1.02)		0.51 (-0.06, 1.08)	
**TDF exposure**				
No exposure	ref	0.865	ref	0.831
< 4 years	-0.13 (-0.75, 0.49)		-0.13 (-0.74, 0.48)	
4 years +	0.01 (-0.83, 0.86)		0.25 (-0.59, 1.08)	

The adjusted model included the following exposures: pubertal status, weight for age z-scores, socio-economic status, orphan status, calcium intake, age at ART initiation and viral load. ART-Anti-retroviral therapy, TDF- tenofovir disoproxil fumarate. CI-confidence interval SMD: Skeletal maturity deviation calculated as the difference between bone age and chronological age; negative values reflect lower bone age than chronological age (delay in skeletal maturation) *mean SMD for each category

## Discussion

We report results from the first study to our knowledge to determine skeletal maturation in the children with HIV in Southern Africa. Underweight and having HIV were strongly associated with delayed skeletal maturation. The delay in skeletal maturity was more marked in the older children who were at earlier Tanner stages. Living with HIV infection and being underweight were strong predictors of skeletal maturity deviation in both males and females. In CWH, later age at ART initiation predicted greater delay in skeletal maturation in females [31].

Similar to this study, a delay in skeletal maturation has been reported in other studies in black African HIV-negative children and adolescents [31–33]. In one, a slightly older cohort of black South African males aged 13–21 years, BA was on average 0.5 years younger than CA [33]. In another Malawian study, CA ranged between 2 and 28 years and there was a high negative average SMD of approximately 1.6 years for both males and females [31]. In a comparison of black and white children in South Africa, black males matured later than white males by 6 months [34]. In contrast, a systematic review of studies of black children, age 0–18 years, living in high income countries showed they have advanced BA relative to CA [35]. The differences in skeletal maturation between black children in Africa and those in high income countries may be explained by the optimal environmental conditions in terms of better access to healthcare and nutrition which are likely to contribute to achieving better genetic potential, all of which play a key role in the skeletal development of children.

Importantly living with HIV was a key predictor of SMD independent of other factors in this cohort such that CWH were less skeletally mature by over 6 months on average, compared to HIV-negative children, despite being established on ART. Older CWH were more likely to be in earlier Tanner stages, with chronologically older CWH in earlier Tanner stages having the more negative SMD. It is possible these children will experience some ‘catch-up’ skeletal maturation on initiation of puberty, which may be more rapid in duration than individuals who are more advanced in maturation at the same age. The 6-month delay is less than that seen in Brazilian and Indian CWH on ART, aged 5–11 and 8–14 years respectively, who had an SMD of over one year, though these studies were smaller and did not include older adolescents, nor in India a comparator group [10, 11].

Age at ART initiation was a predictor of SMD in both males and females highlighting the importance of early initiation of ART. Similarly, a longitudinal analysis of skeletal maturation in a 4-year follow-up study of Brazilian children [11] reported reduced SMD in CWH starting ART early. These findings support WHO recommendations to start ART upon HIV diagnosis regardless of CD4 count [36, 37]. As 80% of Zimbabwean children (0–14 years) have access to ART [1], delayed ART initiation is still a reality [1]. Our study did not show an association between TDF use and SMD, even though TDF has been associated with bone deficits in density and strength (i.e. bone accrual) in the same population [3, 6], potentially reflecting a direct effect of TDF on bone accrual rather than on puberty, or an underpowered analysis [38, 39].

Underweight children, living with or without HIV, were delayed in BA by over a year compared to those not underweight, consistent with a study in India where BA was measured in 100 underweight children. Low calcium intake was associated with delays in skeletal maturation in male CWH highlighting the importance of nutrition in growth and development. In contrast, there was no relationship found between BMI and skeletal maturation in Iranian and Malawian children aged 6–15 years and 2–28 years respectively [31, 40]. The Malawian study used Greulich Pyle to assess BA which we have found to be less biased by age and less precise than Tanner Whitehouse 3 method [21]. In the South African Birth to Twenty Bone Health Study [41], being heavier and taller at age two years in males and having greater lean mass and having entered puberty in females were associated with more advanced development at age 9–10 years, which is in general agreement with the current findings. Early life environmental exposures and growth measures were not measured in the current study but the importance of a healthy weight and association with less delay in development suggests a lifelong effect of weight on development, consistent with previous observations.

The strengths of this study are having a comparator group of HIV negative children, and the study sample was a likely representation of children living in Harare. Another strength is the use of the TW3 method instead of the GP method which was shown to be less valid in our previous work [21]. The cross-sectional nature of our study means causality cannot be implied. There were indications that SMD was associated with age at ART initiation and viral load in boys with HIV, and pubertal status in girls with HIV; however, the relatively small sample size in CWH and in the later Tanner stages may explain this.

## Conclusion

In conclusion perinatally acquired HIV infection and being underweight were independently associated with a delay in skeletal maturation in both the boys and the girls. Given the consequences of delayed development on final height and subsequent outcomes, longitudinal studies are needed to determine the implications of a delayed development on later health in children living with and without HIV.

### Electronic supplementary material

Below is the link to the electronic supplementary material.

Supplementary Material 1

## Data Availability

Anonymised research data will be made available for sharing on the London School of Hygiene & Tropical Medicine (LSHTM) open access data repository (LSHTM Data Compass). Email: Tsitsi.Bandason@lshtm.ac.uk.

## Abbreviations

ART: Anti-retroviral therapy
BA: Bone age
CA: Chronological age
CWH: Children living with HIV
SMD: Skeletal maturity deviation
