# Supplementary material for: The impact of HIV infection on skeletal maturity in peripubertal children in Zimbabwe: a cross-sectional study
Source: BMC Pediatr. 2024 Jul 27;24:480. doi: 10.1186/s12887-024-04965-y (PMC11282653; doi:10.1186/s12887-024-04965-y)
Supplement: Supplementary file 1 — Supplementary Material 1 [file 12887_2024_4965_MOESM1_ESM.docx]

**Supplementary Table 1**. Demographic and anthropometric characteristics in children living with HIV

|  | **Males** | **Females** | **p-value** |
| --- | --- | --- | --- |
|  | n=145 | n=126 |  |
| **Socio-demographics** |  |  |  |
| Chronological age (years), mean (SD) | 12.6 (2.5) | 12.3 (2.4) | 0.200 |
| Socio-economic status, n (%) |  |  | 0.260 |
| Group 1: low | 46 (31.7) | 29 (23.0) |  |
| Group 2: middle | 48 (33.1) | 44 (34.9) |  |
| Group 3: high | 51 (35.2) | 53 (42.1) |  |
| Orphanhood: One or both parents dead n (%) | 59 (42.8) | 52 (42.6) | 1.000 |
| **Anthropometry** |  |  |  |
| Height (cm), mean (SD) | 139.9 (12.3) | 139.2 (12.9) | 0.650 |
| Height for age z-score, mean (SD) | -1.7 (1.1) | -1.5 (1.1) | 0.057 |
| Stunting (height for age z-score<-2), n (%) | 52 (35.9) | 34 (27.0) | 0.15 |
| Weight (kg), mean (SD) | 35.5 (17.5) | 35.1 (13.5) | 0.83 |
| Weight for age z-score, mean (SD) | -1.6 (1.2) | -1.3 (1.2) | 0.023 |
| Underweight (weight for age z-score<-2, n (%) | 47 (32.4) | 27 (21.4) | 0.055 |
| BMI (kg/m2), mean (SD) | 16.5 (1.4) | 17.2 (2.4) | 0.010 |
| BMI for age z-score, mean (SD) | -0.8 (0.9) | -0.6 (0.9) | 0.079 |
| Wasting (BMI for age z-scores<-2, n (%) | 13 (9.0) | 8 (6.3) | 0.500 |
| **Pubertal status** |  |  | 0.16 |
| Tanner I | 54 (39.7) | 53 (44.2) |  |
| Tanner II | 37 (27.2) | 18 (15.0) |  |
| Tanner III | 21 (15.4) | 27 (22.5) |  |
| Tanner IV | 19 (14.0) | 18 (15.0) |  |
| Tanner V | 5 (3.7) | 4 (3.3) |  |
| **Lifestyle factors** |  |  |  |
| Physical activity level, n (%) |  |  | 0.27 |
| *Low, <600 MET mins/week* | 46 (31.7) | 29 (23.0) |  |
| *Moderate, 600-3000 MET mins/week* | 33 (22.8) | 30 (23.8) |  |
| *High, >3000 MET mins/week* | 66 (45.5) | 67 (53.2) |  |
| Daily vitamin D intake, n (%) |  |  | 0.53 |
| *Very low, <4.0 μg/day* | 26 (17.9) | 23 (18.3) |  |
| *Low, 4.0-5.9 μg/day* | 96 (66.2) | 89 (70.6) |  |
| *Moderate, 6.0-8.0 μg/day* | 23 (15.9) | 14 (11.1) |  |
| Daily calcium intake, n (%) |  |  | 0.93 |
| *Very low, <150 mg/day* | 52 (35.9) | 44 (34.9) |  |
| *Low, 150-299 mg/day* | 29 (20.0) | 28 (22.2) |  |
| *Moderate, 300–450 mg/day* | 64 (44.1) | 54 (42.9) |  |
| **HIV Characteristics** |  |  |  |
| Age at diagnosis, n (%) |  |  | 0.70 |
| *<4 years exposure* | 69 (59.0) | 63 (62.4) |  |
| *4-7.9 years* | 36 (30.8) | 25 (24.8) |  |
| *8-12 years* | 8 (6.8) | 10 (9.9) |  |
| *>12 years* | 4 (3.4) | 3 (3.0) |  |
| Age at ART initiation, n (%) |  |  | 0.31 |
| *<2 years* | 38 (26.2) | 36 (28.6) |  |
| *2-3.9 years* | 38 (26.2) | 32 (25.4) |  |
| *4-8 years* | 49 (33.8) | 32 (25.4) |  |
| *>8 years* | 20 (13.8) | 26 (20.6) |  |
| ART duration, n (%) |  |  | 0.38 |
| *2-6 years* | 26 (22.2) | 27 (26.7) |  |
| *6-10 years* | 68 (58.1) | 61 (60.4) |  |
| *>10 years* | 23 (19.7) | 13 (12.9) |  |
| CD4 count, <500 cells per μL | 32 (22.5) | 19 (16.2) | 0.21 |
| Viral load, >1000 RNA copies per ml | 28 (20.9) | 24 (20.3) | 1.000 |
| Tenofovir exposure |  |  | 0.98 |
| *No tenofovir* | 79 (67.5) | 70 (69.3) |  |
| *<4 years exposure* | 24 (20.5) | 20 (19.8) |  |
| *4 years+ exposure* | 14 (12.0) | 11 (10.9) |  |
| **Bone age measures** |  |  |  |
| Bone age, mean (SD) | 11.2 (2.3) | 11.2 (2.4) | 0.97 |
| Skeletal maturity deviation, n (%) | -1.4 (1.4) | -1.1 (1.4) | 0.029 |
| Skeletal maturity delay, n (%) | 45 (31.0) | 26 (20.6) | 0.12 |

*Student t-tests conducted on continuous variables and chi-squared tests on categorical variable.* ***MET -*** *multiples of the resting metabolic rate* ***SD****- Standard deviation. Skeletal maturity deviation-difference between bone age and chronological age. Skeletal maturity delay: skeletal maturity deviation ≤ 2 years*
